# Proteomic comparison of the organic matrices from parietal and base plates of the acorn barnacle *Amphibalanus amphitrite*

**DOI:** 10.1098/rsob.230246

**Published:** 2024-05-29

**Authors:** Janna Schultzhaus, Judson Hervey, Kenan Fears, Christopher Spillmann

**Affiliations:** ^1^ Center for Bio/Molecular Science & Engineering, Naval Research Laboratory, Washington, DC, USA

**Keywords:** acorn barnacle, mass spectrometry, cuticle, chitin binding, cement proteins

## Abstract

Acorn barnacles are efficient colonizers on a wide variety of marine surfaces. As they proliferate on critical infrastructure, their settlement and growth have deleterious effects on performance. To address acorn barnacle biofouling, research has focused on the settlement and adhesion processes with the goal of informing the development of novel coatings. This effort has resulted in the discovery and characterization of several proteins found at the adhesive substrate interface, i.e. cement proteins, and a deepened understanding of the function and composition of the biomaterials within this region. While the adhesive properties at the interface are affected by the interaction between the proteins, substrate and mechanics of the calcified base plate, little attention has been given to the interaction between the proteins and the cuticular material present at the substrate interface. Here, the proteome of the organic matrix isolated from the base plate of the acorn barnacle *Amphibalanus amphitrite* is compared with the chitinous and proteinaceous matrix embedded within *A. amphitrite* parietal plates. The objective was to gain an understanding of how the basal organic matrix may be specialized for adhesion via an in-depth comparative proteome analysis. In general, the majority of proteins identified in the parietal matrix were also found in the basal organic matrix, including nearly all those grouped in classes of cement proteins, enzymes and pheromones. However, the parietal organic matrix was enriched with cuticle-associated proteins, of which *ca* 30% of those identified were unique to the parietal region. In contrast, *ca* 30–40% of the protease inhibitors, enzymes and pheromones identified in the basal organic matrix were unique to this region. Not unexpectedly, nearly 50% of the cement proteins identified in the basal region were significantly distinct from those found in the parietal region. The wider variety of identified proteins in the basal organic matrix indicates a greater diversity of biological function in the vicinity of the substrate interface where several processes related to adhesion, cuticle formation and expansion of the base synchronize to play a key role in organism survival.

## 1. Introduction

The life cycle of the acorn barnacle, a marine arthropod, consists of an exploratory larval stage followed by surface settlement and metamorphosis into a sessile adult which is protected and encapsulated within a calcareous shell. Barnacles rely on a permanent proteinaceous adhesive during settlement and growth. As a primarily colonizing organism, barnacles can be found on a wide range of submerged surfaces in the marine environment, including manmade materials and infrastructure where unwanted biological growth often has a deleterious effect. Significant efforts to develop environmentally friendly and targeted anti-fouling coatings remain a priority [1,2], yet barnacles continue to be one of the most problematic hard macrofoulers [3]. For example, on large ships increased operational and maintenance costs are incurred by the elevated drag forces and increased fuel expenditure [4]. Understanding barnacle adhesion processes to develop more effective antifouling coatings has spurred research into characterizing the macromolecules present at the barnacle–surface interface. Early efforts led to the identification of several cement proteins [5–7], so named because they were first identified in the basal material and, therefore, thought to have a role in adhering the barnacle to their substrate. The development of improved sample processing techniques, mass spectrometry analysis [8,9] and greater transcriptomic and genomic information [10,11] have culminated in the identification of *ca* 90 proteins in the adhesive proteome of the acorn barnacle *Amphibalanus amphitrite* [8,9,12]. These efforts, combined with the relative ease of *A. amphitrite* husbandry in the laboratory environment, have made this species a model organism for antifouling research [3]. From a macromolecule perspective, the cement proteins have received the majority of attention. These proteins are unique to barnacle species and likely have some role in their tenacious adhesion, including low solubility in water and sequence patterns that drive nanofibril formation [8,13–15]. The growing body of work has yet to provide a conclusive explanation of the mechanism underlying barnacle adhesion, which appears to be distinct from adhesive chemistries identified in other marine organisms [16–18], though several theories have been proposed [19–22].

Understanding barnacle adhesion is complicated by the active and often obscured biological processes that occur at the substrate interface. During settlement, cyprids release a permanent adhesive composed of lipids, proteins and chitin [23,24] through their antennae. The attached cyprid of acorn barnacles undergoes metamorphosis and begins growing against the surface while forming a calcareous shell. Observation of the leading edge of growth in juvenile barnacles has highlighted the biological complexity of this region [13,14,25,26]. Columnar cells anchor an active layer of epidermal cells to the parietal (side) shell, placing these cells within microns of the surface interface. The epidermal cells likely produce the cuticle and new circumferential ducts during growth [26,27]. The duct system has been theorized to deliver secretions from cement glands to the surface interface [28,29], yet this idea is contradicted by observations that the adhesive material, comprised protein fibrils, was observed at the interface in advance of new ducts [26]. In fact, proteins and lipids are transferred to the surface as the new cuticle expands, suggesting that the epidermal cells may be their point of origin. Near the epidermal cells, but extending beyond the leading edge of the barnacle, is a hydrophobic fluid rich in oxidative species. This material clears microorganisms and appears to prepare the surface for growth and adhesion of the barnacle base [26].

As the barnacle base grows, it is accompanied by an organic matrix composed of proteins and carbohydrates which, in some acorn barnacle species, is beneath and proximal to a calcified base plate. The matrix facing the substrate interface is protein rich [30], and this direct contact may influence the development of the proteinaceous adhesive. While the importance of the interaction between the surface and the outer protein-rich portion of the biointerface has been examined, protein–carbohydrate bonding likely has a critical but less scrutinized role, having received far less attention despite the fact that both the adult and larval adhesion processes have been theorized to have evolved from ancestral cuticle secretion processes [31]. The observation that chitin is an integral component of the cyprid adhesive, and that a specific protein interacts with the chitin [24], provides supporting evidence for this theory. Other molecular evidence, such as increased levels of reactive carbonyl groups in the adhesive, suggest protein–protein and protein–carbohydrate interactions are likely prominent in the cuticle [26]. Further supporting evidence is the abundance of potential *N-* and, to an even greater extent, *O-*linked glycosylation sites on several proteins identified in the barnacle cement [12].

Proteomic analysis of the adhesive of acorn barnacles has mainly considered the proteins present in the hydrated, rubbery adhesive [8,9,12], as the collection and processing of this material is much more feasible than the thin layer of hardened adhesive formed by acorn barnacles on substrates with high surface energy. Here, the focus of proteomic analysis is shifted to the organic matrix present at the basal region for comparison with a corresponding organic matrix along the parietal (side) shell of *A. amphitrite*. Development of a method for collection of the basal organic matrix regardless of adhesive morphology would aid to standardize the types of experiments and analysis for inter-species comparisons. Of particular interest are cuticle-related proteins and barnacle cement proteins, both of which likely have a role in the stability of the calcareous base and its adhesion to the substrate. It is noteworthy that proteomic analysis identified several cuticular-related proteins in stalked barnacle adhesive [32] yet these proteins have not been identified to the same extent in the rubbery adhesive of acorn barnacles [12]. The results may stem from inter-species differences, variability in sample collection and processing, and the quality of the databases used to search against, all of which highlight the challenges when comparing proteomes. Considering all arthropod cuticles contain a diversity of proteins [33–35], we compare the proteins extracted and identified in the organic matrices from two distinct regions of the acorn barnacle *A. amphitrite*—the organic matrix below the base plate and the matrix within the upper regions of the parietal plates. While there is significant overlap in the protein profile of both, notable differences exist and are discussed in the context of potentially related functions, including how the basal region of barnacles may be adapted to promote adhesion.

## 2. Methods

### 2.1. Barnacle husbandry and sample collection

**Figure 1.**
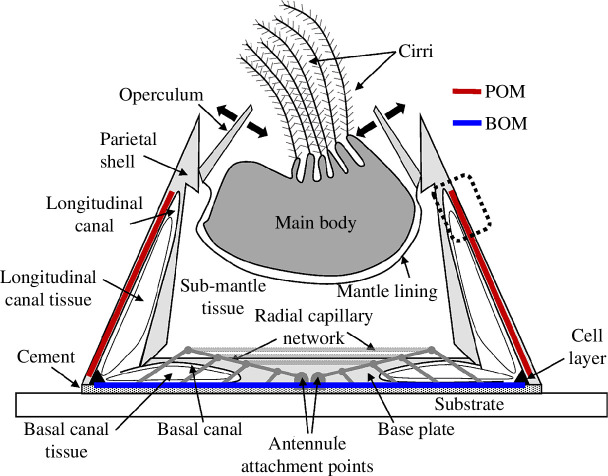
Simplified scheme of barnacle sagittal section highlighting several components, including those factoring into the current work. Red and blue lines represent the POM and BOM, respectively. The dotted box (right-hand side) approximates the region where the POM was collected.

Cyprid *A. amphitrite* were settled on silicone-coated panels at the Duke University Marine Laboratory, and husbandry was performed as previously described [36]. Adult barnacles were gently pushed off the panels, internal soft tissue was removed, and intact shells were stored at –80°C. Upon thawing, intact shells were subjected to two 15 min ice sonication baths in PBS to remove the remaining soft tissue. Shells were rinsed with water and incubated in decalcification buffer (0.5 M EDTA, 2.5% TFA, protease inhibitors (Halt Protease and Phosphatase Inhibitor Cocktail, Thermo Scientific, Waltham, MA, USA) in water) overnight until completion. The cuticular and protein material from the parietal plate organic matrix (POM) and base plate organic matrix (BOM) were separated from one another and the remaining canal tissue embedded within the shells. The POM was collected from the upper region of the parietal plate to optimize differentiation of the material from the BOM (see figure 1). The matrices were washed three times with optima grade water (Thermo Scientific, Waltham, MA, USA), flash frozen with liquid nitrogen, and ground via mortar and pestle. The ground matrices were dried via Speed-Vac (Savant/ThermoScientific, Waltham, MA, USA). Proteins were extracted following a protocol for insect cuticle protein extraction [33,34]. Samples were incubated overnight at room temperature in hexafluoroisopropanol (HFIP). The HFIP was removed via Speed-Vac and samples were incubated in 100 µl of 0.1% Rapigest (Waters, Milford, MA, USA) in 100 mM ammonium bicarbonate (ABC) overnight in a Thermomixer (Thermo Scientific, Waltham, MA, USA) (RT, 1000 rpm). Samples were then boiled at 100°C for 5 min, centrifuged at 21 000*g* for 10 min, and transferred to a new tube. Protein quantification was performed via Nanodrop. Disulfide bonds were treated with 10 mM dithiothreitol (DTT, 30 min at 60°C and 1000 rpm), 30 mM iodoacetamide (30 min in the dark at RT and 1000 rpm) and 10 mM DTT (30 min in the dark at RT and 1000 rpm). In total, 10 µg aliquots of the extracted proteins were digested with 1 : 25 ratio trypsin (Promega, Madison, WI, USA) and 1 : 25 ratio LysC (Wako, Chesterfield, VA, USA) for 18 h at 37°C and 1000 rpm. Samples were acidified with trifluoracetic acid to a final concentration of 0.5% and incubated for 45 min at 37°C and 1000 rpm. Samples were centrifuged at 13 000 rpm for 10 min, and the supernatant was transferred to a fresh vial and dried via Speed-Vac.

### 2.2. Liquid chromatography and tandem mass spectrometry

Liquid chromatography–tandem mass spectrometry (LC-MS/MS) was performed with an Ultimate 3000 LC system (Thermo Scientific, Waltham, MA, USA) coupled to an Orbitrap Fusion Lumos Tribrid mass spectrometer.

The LC system was configured for one-dimensional nanoflow separations with online desalting. Dried peptides were resuspended to a concentration of 0.1 ng µl^−1^ in 0.1% formic acid and 100 ng was injected via the autosampler and concentrated onto a trap column (PepMap 100, C_18_, 300 µm ID × 5 mm, 5 µm, 100 Å) with the loading pump at a flow of 2% solvent B (0.1% formic acid in acetonitrile) and 98% solvent A (0.1% formic acid in water) at 5 µl min^−1^. After desalting for 3 min, the flow was diverted inline at 300 nl min^−1^ for separation across a reverse phase analytical column (Acclaim PepMap RSLC, 75 µm ID × 150 mm, C_18_, 2 µm, 100 Å) for a total duration of 120 min. A two-step gradient of increasing solvent B (18% over first 80 min, followed by an increase of 60% over 15 min) was used to elute peptides from the analytical column. The LC separation was coupled to a Nanospray Flex Ion Source of an Orbitrap Fusion Lumos Tribrid mass spectrometer operating in data-dependent acquisition mode. The Orbitrap detector was set to survey precursor ions at a resolution of 120K across a mass-to-charge (*m*/*z*) range of 400–1600. Maximum injection time was 50 ms and the automatic gain control (AGC) target was 400 000. The most intense precursor ions with charges of 2–7 were fragmented using HCD (higher-energy collisional dissociation), and ions were excluded for 30 s from subsequent MS/MS submission via dynamic exclusion. The ion trap was used as the MS/MS detector with 35 ms injection time and an AGC target of 10 000.

### 2.3. Data analysis

Peptide spectrum matching (PSM) between tandem MS data and reference protein sequences was performed with MaxQuant (version 1.6.10.46) [37] and Mascot Server (version 2.8.2). MaxQuant identifications were assigned to a manually curated *A. amphitrite* protein database composed of the protein sequences originating from the open reading frames of a draft genome sequence [11] (GenBank record GCA_009805615.1, annotated via the NCBI Eukaryotic Genome Annotation Pipeline [38]) and additional protein sequences identified through transcriptomics [10] which were missing from the draft genome, as previously described [12]. Common protein contaminant sequences (e.g. human keratins, trypsin, BSA, etc.) arising from sample handling and processing were included during PSM. Protein accessions containing the text strings cont* or sp*, along with their accompanying protein description, denote a protein as a contaminant identified as an artefact of sample processing for LC-MS/MS. Default MaxQuant parameters were used for PSM identification with the exception of the following parameters listed below: cysteine carbamidomethylation (fixed); methionine oxidation and acetylated protein *N-*term (variable); and label-free quantification (LFQ), match between runs features were enabled. Results of MaxQuant searches are described in further detail and used for subsequent primary sequence analysis. The electronic supplementary material describes primary data extraction, PSM search parameters, supplemental Mascot search results and entries in the predicted *A. amphitrite* reference proteome used for PSM, as previously described [39]. *Amphibalanus amphitrite* protein functions and descriptions were assigned via the NCBI Eukaryotic Genome Annotation Pipeline [11,38], based in part upon inference from primary sequence homology [40].

MaxQuant grouped PSMs into proteins and assigned LFQ intensities for each identification. MaxQuant PSM assignment, output as proteinsgroups.txt, was imported into R (version 4.0.5) and the DEP package [41] computed differential protein abundance from LFQ intensities. Data were filtered to exclude proteins with excessive missing values (filt = 1). Missing values were imputed using a mixed imputation method with missing not at random proteins (defined when proteins were missing from all replicates in at least one condition) imputed using a zero method (all NA cells replaced with 0 values), and missing at random proteins were imputed using the BPCA algorithm. The statistical cut-off for significance was set at *α* = 0.05 and log_2_ fold change of 1.0. All plots were generated with DEP, VennDiagram [42] or treemapify command of the ggplot2 [43] packages. *N-* and *O-*glycosylation sites were predicted using MusiteDeep [44]. The resulting inventory of protein identifications was subjected to extensive primary sequence analysis for functional inference.

MS data (primary mass spectrometry data, intermediate peak lists and formatted results) were deposited in the Mass Spectrometry Interactive Virtual Environment (MassIVE, http://massive.ucsd.edu), a member of the ProteomeXchange Consortium. The dataset was assigned MassIVE accession MSV000093654, doi: https ://doi.org/doi:10.25345/C53N20R13. A mirrored dataset was deposited in ProteomeXchange (PDX).

### 2.4. Electron microscopy

Electron microscopy (EM) imaging was completed as described previously [9] where both a partial demineralization protocol [45] and a similar protocol with no demineralization were implemented. For demineralization, an acid solution was added to the epoxy-embedded and polished cross sections and rinsed after 30 s. Images of both the native calcified samples and demineralized samples were taken from the same slice using a Leo field emission secondary electron microscope (Carl Zeiss Microscopy).

## 3. Results

### 3.1. Organic matrix isolation

**Figure 2.**
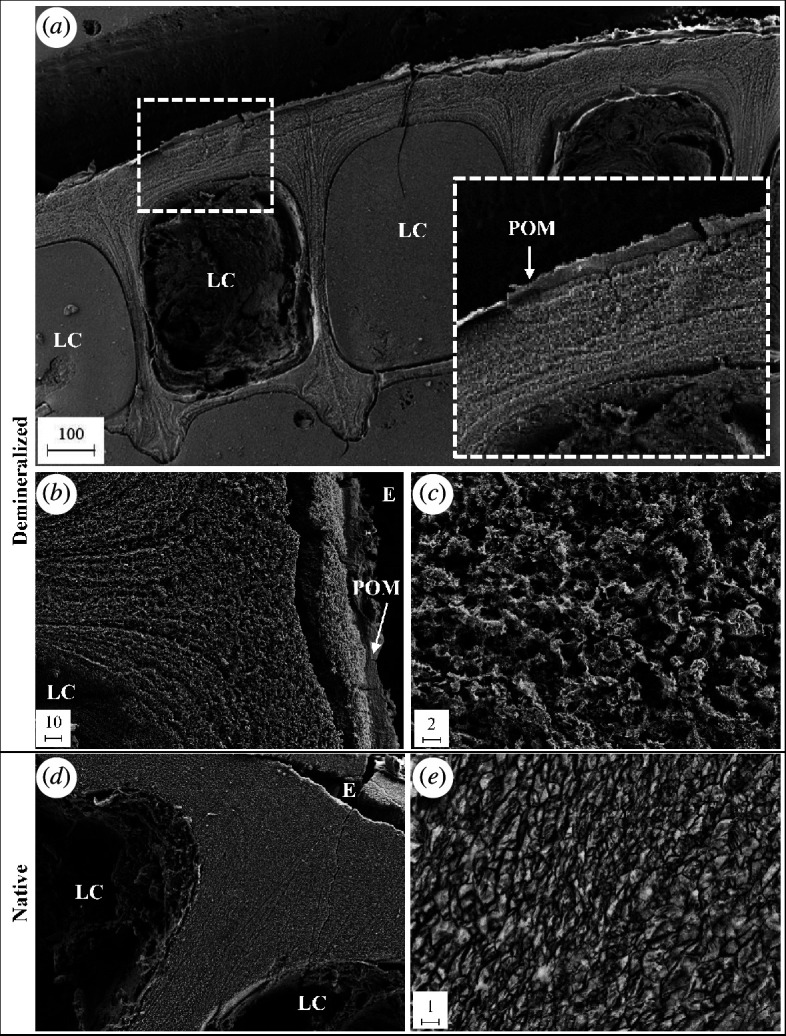
Electron microscopy of *A. amphitrite* parietal plates. Adjacent sections about 3 mm from the barnacle base were either demineralized prior to imaging (*a–c*) or imaged in their native calcified state (*d–e*). (*a*) Magnification at 100× of the demineralized samples shows the organic matrix located at the edge of the parietal plate and emphasized in the inset. No organic membrane embedded within the shell is visible at (*b*) 1000× or (*c*) 5000×. (*d*) Image of native sample where the internal structure of the parietal plate appears homogeneous (1000× mag). (*e*) Calcite crystals within parietal shell are apparent at 10 000×. All scale bars are in micrometres. E = edge; POM = parietal organic matrix; LC = longitudinal canal.

The organic matrices from the basal and parietal regions of the barnacle were collected for downstream processing and analysis. Careful dissection and preparation of POM and BOM samples were performed to minimize cellular and cross-sample contamination. A schematic representation of a sagittal section of an acorn barnacle highlighting some of the relevant components and sample collection sites is shown in figure 1. The soft tissue (main body and sub-mantle tissue) was removed from inside the shells of adult barnacles displaced from silicone panels. The entire shell was then subjected to decalcification; the mid-section of the parietal plate decalcified faster than the upper portion near the operculum (figure 1). The partially decalcified parietal shells and the base plate were separated and, after cleaning the remaining parietal plate material away from the base plate, decalcification was completed for both materials separately to minimize cross-sample contamination. Upon complete decalcification, the embedded organic matrices and canal tissues were released. Canal tissue is the soft tissue that fills the open canals of both the parietal and base plates (figures 1 and 2; [46]) and was removed from the organic matrices. While the POM visually appeared to be composed of a uniform cuticle-like material, the BOM contained more distinctive features. The substrate face of the BOM, which is known to be protein rich [13,30], appeared as a thick, rubbery adhesive. In addition, the BOM contained both an embedded, inseparable radial capillary network (figure 1; [14,25]) not present in the POM and a thin epithelial cell layer at the leading edge [26] that was unable to be removed manually during dissection (represented as black triangles at the leading edge of the barnacle base in figure 1). These parietal and basal materials were processed for subsequent proteomic analysis.

### 3.2. Electron microscopy of the parietal plates

To visualize the organic matrix adjacent to the parietal shell of *A. amphitrite*, EM was performed on side shells approximately 3 mm from the base. In partially demineralized sections, an organic layer is apparent at the edge of the parietal plate and another layer lining the longitudinal canals (figure 2*a,b*); the canals are readily apparent structures embedded vertically within the side shell [46]. Images of the central portion of the parietal plates, whether the demineralized (figure 2*b,c*) or native samples (figure 2*d,e*), indicate this region is a consistent layered inorganic material, i.e. calcified shell. Magnified images of the shell confirm this striated texture, presumably either residual or fully formed calcite crystals (figure 2*c,e*). The structure on the outer edge of the shell (figure 2*a,b*) is the POM isolated for proteomic analysis. The dashed box in the upper right of figure 1 represents the relative region from which the POM was collected, highlighting the effort to isolate the organic matrices from one another.

### 3.3. Organic matrix proteome description

**Figure 3.**
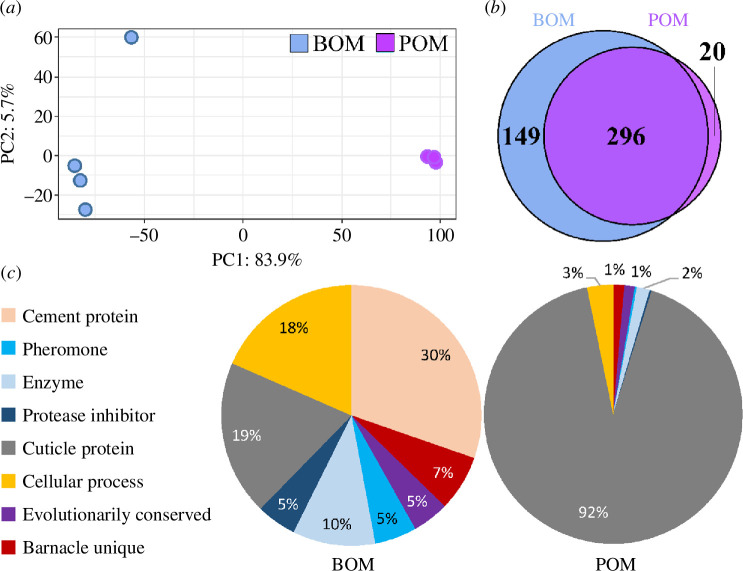
Proteomic comparison of the base and parietal plate organic matrices of *A. amphitrite*. (*a*) Principal component analysis (PCA) of the total proteome of each replicate shows tighter clustering of the POM samples but distinct separation of BOM and POM samples along the PC1 axis. (*b*) Venn diagram showing the overlap in proteins identified in the BOM and POM. (*c*) Pie charts depicting the intensity values (LFQ) of each protein category in the BOM (left) and POM (right).

In general, the proteomic profile of the organic matrices are distinct despite a significant overlap in the number of proteins identified in each. Quality assessment of the variability between samples indicates tight clustering of the POM with a more dispersed arrangement of the BOM samples in a principal component analysis (PCA) (figure 3*a*). The BOM and POM sample sets separate clearly along the PC1 axis, which explains 84% of the total variability. A similar number of proteins were identified in each biological replicate per sample type after filtering proteins with missing values (filt = 1): 300 ± 10 proteins per BOM sample (*n* = 5); 220 ± 10 proteins per POM sample (*n* = 4). Overall, more total proteins were identified in the BOM samples (445) than in the POM samples (316). Of all those identified, 296 (64%) were shared between the two sample types. In the POM, 20 unique proteins were identified, whereas 149 proteins were unique to the BOM (figure 3*b*; electronic supplementary material, file S1).

Each identified protein was placed into one of eight categories to gain perspective on the potential functional differences between the organic matrix samples: cement proteins, pheromones, enzymes, protease inhibitors, cuticle proteins, cellular process proteins, barnacle unique proteins and evolutionarily conserved proteins. The cement proteins are those first identified in the barnacle adhesive layer and are thought to underlie the adhesion process or compose the adhesive bulk [6,8,20,47]. These are unique to barnacles, with no homologues identified in any other taxa and do not contain any conserved domains that indicate their biological function. Many of the cement proteins exhibit sequence similarity to one another, forming multiple cement protein families. Barnacle pheromones consist of α-macroglobulin pheromones (settlement-inducing protein complex: SIPC; MULTIFUNCin: Multi) [48–50] and waterborne settlement pheromones (WSP) [51,52]. Enzymes, protease inhibitors, cuticle proteins and proteins involved with cellular processes were identified via annotation, conserved domains and gene ontology (GO) terms. The cellular process category contains proteins that are either localized or function within the cell, in the cell membrane or in the extracellular matrix. Evolutionarily conserved proteins refer to those with homologues identified in organisms across other taxa and often contain conserved domains or annotations that imply a biological function but cannot be definitively determined to function intracellularly. Barnacle unique proteins are defined as those unique to barnacle species, yet have not received the same attention by researchers as cement proteins. They do not often form families based on sequence similarity and may contain conserved domains.

**Figure 4.**
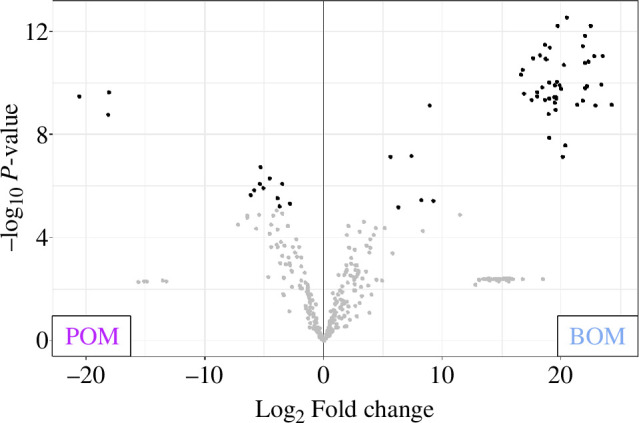
Volcano plot displaying proteins with significantly different levels of abundance (black points) in the BOM and POM samples based on a greater than twofold change and *p*-value = 0.05 cut-offs.

The proportion of the total intensity of each protein category highlights the distinct profiles of the BOM and POM (figure *3c*). Cement proteins make up the largest portion of the BOM (30%), with cuticle proteins (19%) and proteins involved with cellular (18%) or enzymatic (10%) processes following. These four categories comprise about three-quarters of the total abundance of BOM samples. The other proteins identified were distributed among the remaining categories (pheromones, protease inhibitors, evolutionarily conserved and barnacle unique). In stark contrast, the vast majority of the POM composition was identified as cuticle proteins (92%). The cellular process, enzyme and evolutionarily conserved and barnacle unique protein categories each comprise *ca* 1–3%; the remaining categories had less than 1% of the total POM intensity. Of note, cement proteins contribute less than 0.02% of the POM intensity and confirm our previous work demonstrating the presence of cement proteins in regions far removed from the substrate interface [53]. An abundance analysis revealed that 18% (64 of 350 assessed for differences in abundance levels) of the total proteins identified were found to differ significantly between the BOM and POM samples after normalization (figure 4, electronic supplementary material, file S2). The POM samples had 13 proteins with statistically higher abundances, of which eight were cuticle proteins. The more abundant BOM proteins comprised proteins from all categories. Not unexpectedly, 12 cement proteins were found to be more abundant in the BOM samples as well as eight other proteins in the barnacle unique category.

### 3.4. Cement proteins

Cement proteins have been isolated from the adhesive of multiple species of barnacles over the last several decades and were named initially according to their apparent molecular weight (e.g. CP19k) [5–7]. To date, eight *A. amphitrite* cement protein (AaCPs) families, comprised 28 proteins, have been described [8,9,12]. Of these, 24 were identified in the BOM and 10 were identified in the POM (table 1). All cement protein families were identified in the BOM with the exception of AaCP20. It is noteworthy that CP20 proteins have not been observed in multiple proteomic analyses of *A. amphitrite* adhesive, and it has been suggested by Fears *et al*. that this protein family is likely associated with the barnacle baseplate itself [54]. Cement proteins from five families (AaCP19, 43,52, 57 and 100) were identified in the POM.

**Table 1.** Cement protein families identified in barnacle organic matrices.

**f**amily	no. of **m**embers **d**escribed	no. identified BOM	no. identified POM
AaCP19	6	6	2
AaCP20	2	0	0
AaCP34	3	3	0
AaCP43	3	3	3
AaCP52	5	4	2
AaCP57	4	4	1
AaCP100	2	2	2
AaCP105	3	2	0

**Figure 5.**
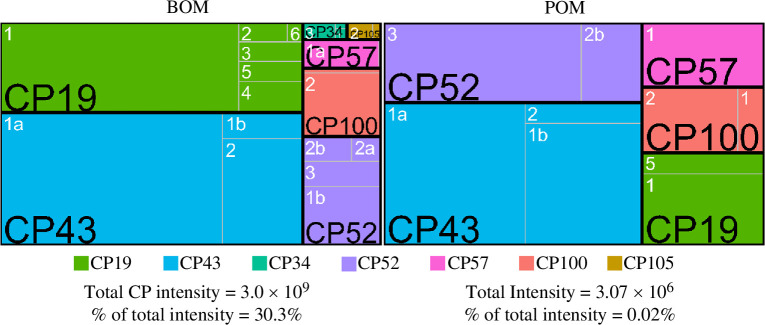
Proportional intensity of each cement protein family and member for the BOM (left) and POM (right) samples. Families are delimited by black lines/text and members by grey lines/white text. Note: the total intensity for all cement proteins varies by three orders of magnitude between BOM (×10^9^) and POM (×10^6^); the total cement protein (CP) intensities and their share of the total sample intensity are shown.

To get a sense of the relative abundance of cement proteins in the BOM and POM, the intensity of each cement protein family and member in relation to the total intensity was examined (figure 5). Individual protein intensity measurements were computed from MaxQuant LFQ values (electronic supplementary material, file S1). The AaCP43 family was the most abundant in the BOM (47%) and POM (44%) samples, with AaCP43A-1 making up the majority in both matrices. AaCP19 and AaCP52 were the next most abundant, with AaCP19 being more abundant in base material (32%) and AaCP52 more abundant in the parietal collections (24%). The AaCP57 and AaCP100 families were the next most abundant, each accounting for 3–9% of the total abundance for both BOM and POM. The AaCP34 and AaCP105 families were minor components of only the BOM (1% for each family). It is noted that the low abundance of these latter families (AaCP34, AaCP57, AaCP100 and AaCP105) may be a reflection of the relatively poor extraction efficiencies achieved with HFIP [9].

While the absolute intensity of the cement proteins is much higher in the BOM samples compared to the POM (×10^9^ versus ×10^6^, respectively), the use of normalization procedures enabled statistical analysis of protein abundance differences between the BOM and POM. Of the identified cement proteins, 12 were found to have significantly higher abundances in the BOM samples (electronic supplementary material, file S3). Four that were not significantly different (AaCP19-6, AaCP57A-2, AaCP57C-1 and AaCP105-1) were not identified in enough replicates for statistical assessment, even though they meet the threshold necessary for identification. AaCP19-5, AaCP43A-1 and -2, AaCP52-3 and both AaCP100 proteins did not differ between the BOM and POM samples.

### 3.5. Cuticle proteins

Beyond the cement proteins, the other prominent class present in the BOM and POM samples were cuticle proteins, which were identified via CutProtFam-Pred [55] and by examination on UniProt. Based on the CutProtFam analysis and the types of chitin-binding domains present, the 64 cuticle-identified proteins were sorted into two families: cuticle proteins with the Rebers and Riddiford (RR) consensus region and peritrophin-type cuticle proteins (CPAP) (table 2). The vast majority were identified as RR, all of which had one (*n* = 50) or two (*n* = 3) CHIT_BIND_RR_2 domains; all were described with the GO term ‘structural constituent of the cuticle’ (Molecular Function, 0042302). Of the 10 CPAP proteins identified, all had at least one copy of the CHIT_BIND_II domains (PS50940) and the GO terms ‘chitin binding’ (Molecular Function, 0008061) and ‘extracellular region’ (Cellular Component, 0005576). The RR cuticle proteins are the most abundant in both the BOM and POM samples, composing greater than 98% of the total cuticle protein intensity. Of note, the POM intensity values are an order of magnitude higher than in the BOM samples. The majority of the identified cuticle proteins had levels of relative abundance between the two sample types. Of those that did have a statistically significant different level, eight were more abundant in the POM and only one in the BOM. Of these nine proteins, eight were identified with the RR consensus sequence. We also note that enzymes involved with chitin metabolism (one chitinase (KAF0301779.1) and three chitin deacetylases (KAF0300422.1, KAF0300493.1 and KAF0311795.1)) were detected in both the BOM and POM samples.

**Table 2.** Cuticle proteins identified in the organic matrices.

**domain**	**no. of** **proteins**	**ave LFQ intensity**	**per cent intensity of** **cuticle proteins**	**per cent of total** **intensity**
BOM				
CPAP^a^	10	6.0 × 10^6^ ± 5 × 10^5^	1.6%	19%
RR^b^	48	3.8 × 10^8^ ± 7 × 10^7^	98.4%	
POM				
CPAP	8	2.7 × 10^7^ ± 2 × 10^6^	0.6%	92%
RR	44	4.5 × 10^9^ ± 5 × 10^8^	99.4%	

^a^ CPAP: cuticular proteins analogous to peritrophins.

^b^ RR: Rebers and Riddiford consensus region.

### 3.6. Categorical differences in unique and differentially abundant proteins

The remaining categories of identified proteins (cellular processes, protease inhibitors, enzymes, pheromones, evolutionarily conserved and barnacle unique proteins) are diverse with numerous potential biological functions. As such, the analysis is limited to summarizing the differences between the two organic matrix samples and a description of proteins not identified in prior analyses.

**Figure 6.**
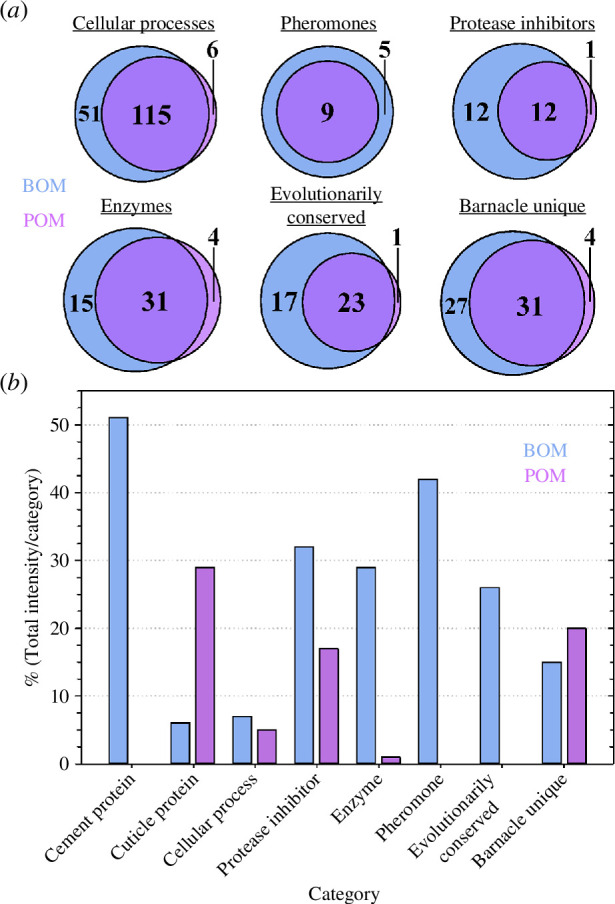
Comparison of protein categories. (*a*) Venn diagrams depicting the overlap in the number of identified proteins in the Cellular Processes, Pheromones, Protease Inhibitors, Enzymes, Evolutionarily conserved and Barnacle unique categories. (*b*) Comparison plot of the percentage of proteins of the total *category* intensity (rather than the total sample intensity) that are unique to a particular matrix *or* with a statistically significant abundance in either matrix.

The BOM samples had a greater diversity and more proteins identified in each category (figure 3, 6*a* table 3). In addition, the absolute intensity values for each protein category were generally higher in the BOM samples (figure 3*c*, table 3). Once the protein levels were normalized with the DEP package to enable statistical analysis (see §2.3), another layer of comparison was applied to better understand the protein profile *within each category* (see electronic supplementary material S4). Figure 6*b* is a bar plot showing the percentage of protein total intensity within each of the eight categories from the BOM or POM that were either (i) unique to a particular matrix or (ii) had a statistically significant abundance in either matrix. For example, in the cement protein category, 51% of the proteins were either unique to the BOM or had a greater abundance in the BOM (and no cement proteins met these criteria in the POM). In contrast, for the cuticle protein category, 29% of the cuticle proteins were either unique or more abundant in the POM; the percentage of the total protein intensity of the cuticle proteins in the BOM was only 6%. From figure 6*b*, it is clear that material assigned in the BOM has a greater percentage of unique or statistically significant abundant total protein intensity within each category apart from the cuticle proteins and, to a lesser extent, with the barnacle unique category. In several instances (cement proteins, pheromones and evolutionarily conserved proteins categories), the percentage of unique or statistically significant abundant proteins was exclusively in the BOM. It is noteworthy that the majority of proteins in each category were either found in both the BOM and POM or did not have a statistically significant difference in abundance. The sole exception is the cement protein category in the BOM, where 49% of the protein intensity was either shared with the POM or did not show a significant difference in abundance. Ultimately, these observations indicate the increased sample heterogeneity of the BOM samples, as noted in figure 3*a* and *c*.

**Table 3.** BOM and POM comparison of six protein categories.

	**BOM**			**POM**			
**category**	**no.** ^**a**^	**ave LFQ** **intensity** ^**b**^	**per cent total** **intensity**	**no.**	**ave LFQ** **intensity**	**per cent total** **intensity**	**no.** **significant** ^**c**^
cellular processes	166	3.7 × 10^8^ ± 2 × 10^8^	18%	121	1.6 × 10^8^ ± 1 × 10^7^	3.2%	10 ↑ BOM 1 ↑ POM
protease inhibitors	24	1.3 × 10^8^ ± 3 × 10^7^	5%	13	1.2 × 10^7^ ± 1 × 10^6^	0.2%	6 ↑ BOM 1 ↑ POM
enzymes	46	2.1 × 10^8^ ± 2 × 10^7^	10%	35	8.0 × 10^7^ ± 1 × 10^7^	1.6%	6 ↑ BOM
pheromones	14	1.1 × 10^8^ ± 2 × 10^7^	5%	9	1.4 × 10^7^ ± 8 × 10^5^	0.3%	2 ↑ BOM
evolutionarily conserved	40	9.1 × 10^7^ ± 7 × 10^6^	5%	24	6.5 × 10^7^ ± 1 × 10^7^	1.3%	7 ↑ BOM
barnacle unique	74	2.3 × 10^8^ ± 9 × 10^7^	7%	51	6.8 × 10^7^ ± 3 × 10^6^	1.4%	8 ↑ BOM 3 ↑ POM

^a^ Number of identified proteins in each category.

^b^ Average intensity (LFQ values ± SE).

^c^ Number of significant proteins per category and how many were more abundant in the BOM or the POM samples (no. ↑ BOM/POM).

Some of the unique and statistically significant proteins are noted. The 31 POM and BOM proteins in the barnacle unique category were not identified in past proteomic analyses of the adhesive [12], indicating these proteins may be novel barnacle cuticle-associating proteins. About half of the intensity of the statistically unique BOM evolutionarily conserved proteins is owing to venom allergen or CAP domain-containing proteins, which have been previously identified in adhesive samples [8,9], although a predicted function has not been hypothesized for these proteins. The statistically unique BOM enzymes consist of several serine proteases, a cysteine peptidase, a peroxidase and a lysyl oxidase, all of which have been previously observed in the adhesive [8,9,56]. Additionally, an *O-*linked-mannose beta-1,2-*N-*acetylglucosaminyltransferase 1 (KAF0289223.1) was identified only in the BOM, lending support that *O-*glycosylation of the adhesive interface proteins may be important [12]. The BOM protease inhibitors are composed of serine protease inhibitors, whey acid proteins (WAP), and peptidase inhibitors. The WAP and peptidase inhibitors each make up *ca* 50% of the total unique BOM protease inhibitor intensity. Lastly, the majority of the unique BOM pheromone intensity is owing to AaMulti-1 (KAF0312386.1; 40% of the total 42% observed).

## 4. Discussion

The barnacle substrate interface is a complex region where the substrate, biomolecules and base plate (in certain species) interact during growth to permanently cement the barnacle in place. Much effort has focused on identifying proteins within the cement [5–9,32,47,57–60]. While these studies have highlighted the importance of base plate mechanics and the interaction between proteins and the surface for adhesion [30,61–64], they have not accounted for another important part of the barnacle base: the cuticular material present at the surface interface. The cuticle is a critical component common among all arthropods. Its role and interaction with proteins and the adjacent base plate have received far less attention than the protein-rich cement, yet it has an important role in the development and expansion of the barnacle base. In this article, we set forth to identify proteins associated with isolated cuticular material, particularly at the barnacle base. We examined the organic matrices of *A. amphitrite*, a model acorn barnacle for antifouling research, from two distinct regions of the animal: the upper portion of the parietal plate (i.e. side shell) and the base of the animal. Using proteomic analysis with the aid of EM, we analysed and compared the proteomic profiles of these two materials. The results expand the knowledge of proteins associated with the basal cuticle and offer insight into the potential specialized features near the adhesive interface of *A. amphitrite*.

Recently, the structure of the parietal plates of *Austromegabalanus psittacus* has been analysed to understand the role of the organic matrix in biomineralization [45,65,66]. In this species of giant barnacle, multiple layers of the organic matrix form in concentric rings within the parietal plates. In *A. amphitrite*, this level of organization is not observed under EM. The cuticular material is only seen at the outer edge of the shell with no evidence of it being embedded within the shell. The lining of the longitudinal canals, which run vertically in parallel with each other within the parietal plates, also likely contains a lining of cuticular material that envelopes the tissue within [46]. The parietal plates of *A. amphitrite* are much thinner than those of *A. psittacus*, which may explain this difference in organic matrix organization. In crustaceans, the cuticle is known to have a role in regulating biomineralization [67]. For the thicker parietal plates present in *A. psittacus*, concentric rings of cuticle layers may have a similar role and be necessary to aid biomineralization.

The proteomics data in the current study support the hypothesis that the organic matrices present at the parietal and base plates of *A. amphitrite* contribute to biomineralization. From an enzymatic perspective, several predicted proteins were annotated as carbonic anhydrases and carboxylesterases; these enzymes are necessary for calcification and have been identified previously in a proteomic analysis of *A. amphitrite* shell [68] as well as tissues involved in calcification in other marine organisms [69]. Additionally, the role of the extracellular matrix, glycoproteins and proteoglycans in biomineralization is well documented in eggshells [70,71], marine invertebrates [69,72] and the barnacle *A. psittacus* [45]. Here, similar annotated proteins in the basal and parietal organic matrices were identified as potential proteoglycans using MusiteDeep [44] as well as others with likely localization in the extracellular matrix. These data suggest the cuticular material associated with the shell, both in the base and parietal plates, has a role in controlling biomineralization processes in *A. amphitrite*. A comparative examination of the base and parietal organic matrices in an acorn barnacle without a calcified base plate would provide greater support for these hypotheses, as the membranous base would likely be lacking proteins important for biomineralization.

While many elements of the base and POM proteomes are similar, noteworthy differences exist. Cuticle proteins make up greater than 90% of the protein abundance of the POM (figure 3*c*). These results were expected, as POM samples were prepared from a region of the barnacle expected to be cuticle rich. The presence of cuticle was expected to influence the protein profile of the BOM and a more diverse protein profile was not surprising owing to adjacent cementing activity, biomineralization of base plate and the general expansive growth of the base with each moult cycle. As such, cuticle proteins contribute only *ca* 20% of the total BOM protein intensity, with the cement protein, cellular process protein and enzyme categories each making up a significant fraction of the total protein intensity. The diversity of the BOM is further accentuated by the 7% of barnacle unique proteins identified for which no biochemical or physiological function has previously been described.

**Figure 7.**
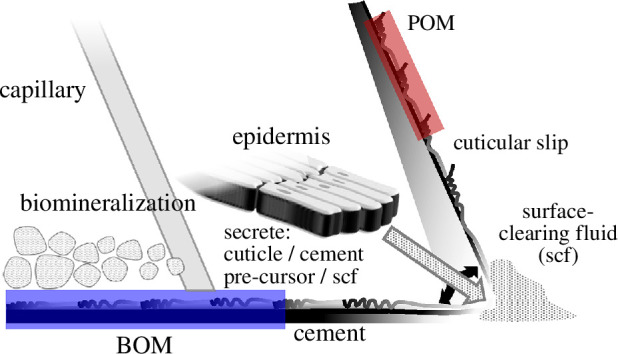
Zoomed scheme of the barnacle basal leading edge. The epidermis (see ‘cell layer,’ figure 1) is depicted secreting cuticular material, surface-clearing fluid (SCF), and (pre-cursor) cement components. Sclerotized cuticle is shown as the POM (red) and BOM (blue) along the parietal shell and base, respectively.

The results presented here underscore the complex nature of the organic matrix, where a clear differentiation in the protein profile is reported for material collected from the base at the substrate interface and the upper side shell of the barnacle *A. amphitrite*. As arthropods, barnacles grow in a cyclic manner via ecdysis, i.e. moulting of their cuticle. For the upper portion of the soft tissue (main body, cirri, etc.; see figure 1), this process is obvious by the presence of the exuviae, i.e. shed exoskeleton. Ecdysis also occurs at the leading edge of the barnacle base with the added layer of complexity from cement deposition and, further removed from the leading edge, biomineralization. At the leading edge of the base, new cuticle extends along the outside of the parietal shell *and* underneath the base. For the former, the Balanomorpha order (which includes *A. amphitrite*) has been shown to form a new cuticle underneath the existing one with the old cuticle expanding, splitting and then being retained as one of a series of cuticular slips on the exterior parietal shell wall [73,74]; a cuticular slip is shown as the POM in figure 2*a,b* and schematically represented in figure 7. The layering of the inorganic features in the EM images indicate the matrix material (and/or a similar material originating from the canal tissue) may also serve as an organic framework for subsequent biomineralization as the shell thickens with moult cycles. Underneath the barnacle at the basal leading edge, confocal microscopy shows existing cuticular matrix at the substrate interface being stretched during radial growth of the base and appearing to partially degrade as a new cuticle forms above it during a moult cycle [14,25,26]. Over several cycles, the result is a series of cuticular annuli along the barnacle base, which was collected and analysed as the BOM. A layer of cells above the cuticle and extending from the leading edge and back towards the centre of the barnacle then provides the building blocks for subsequent biomineralization, with the cuticular material aiding to provide an organic template for expansion of the calcified base plate.

An important issue is the origin of these organic matrices, and the source is likely the epidermis at the basal leading edge of acorn barnacles (figures 1 (black triangles) and 7; [26,73]). As no other prominent cellular features have been observed in this region, this cell layer is responsible for secretion of the cuticular matrix biomolecules. In a more typical insect cuticle, the components include chitin, lipids and proteins [75]. Interestingly, a hydrophobic material has been observed secreting beyond the basal leading edge of *A. amphitrite* during a moult [26]. This material appears to clear the existing microbial biofilm from the substrate as the barnacle prepares to expand its adherent base; this secretion fits as the lipidic or waxy material known to accompany cuticle formation. Furthermore, cement fibrils at basal leading edge have been documented and found to contain proteins in barnacle cement [8,26]. Fears *et al.* showed these nanofibrils are deposited in advance of newly formed ducts [26], which have been attributed to the capillary network through which cement is deposited [76,77].

Collectively, a picture emerges with the basal leading edge epithelium being a key feature to several critical activities: cuticle formation, preparation of the substrate interface for radial growth of the base, cement deposition and supply of an organic matrix for biomineralization. This is schematically depicted in figure 7. Given the presence of cuticular slips along the parietal shell, it is possible that both POM and BOM may have originated from the epithelial layer at the basal leading edge. No known cell layer far up the external side of the calcified parietal shell has been identified to produce a cuticular matrix. (While it is possible that an organic matrix may originate from *inside* the longitudinal canals of the parietal plates, this material was not collected as the POM.) Therefore, it cannot be ruled out that the proteomic differences observed in the POM and BOM may be owing to environmental exposure: the POM is consistently exposed to the external environment while the BOM is protected beneath the barnacle base. Such a conclusion could be supported by the fact that the POM and BOM show a strong overlap in their protein profile (greater than 60%, 296 out of 465) and greater than 95% of the total were identified in the BOM (445 out of 465), see figure 3*b*. Prolonged, i.e. several weeks, exposure of the POM to the external environment, including persistent microbial exposure, would degrade the proteomic profile, while the BOM is in a more insulated and controlled local environment despite the presence of enzymatic cascades associated with cuticle breakdown, sclerotization and cement cross-linking. The model we present poses a challenge to isolate and understand the biochemical production, especially the protein component, of the epithelial layer at the basal edge of *A. amphitrite* and is the subject of ongoing investigation. These findings lead to further questions regarding the location, function and role of proteins and tissues previously attributed to the barnacle adhesion process [53]. Our work also suggests that an important strategy related to controlling barnacle biofouling may lie with compounds in paints and coatings that target and disrupt cuticle formation.

## 5. Conclusion

In comparing the proteomic profile of the organic matrices located in the basal region and along the parietal plates of *A. amphitrite*, significant differences were observed when the data were normalized by the total protein intensity. The overwhelming majority of proteins identified in the POM were cuticle-associated with a significant number unique to the material collected from the parietal region. In contrast, a wide distribution of proteins were identified in the BOM and appear to be representative of the diverse activities found at the basal region of *A. amphitrite* that span cuticle formation, deposition of cement and biominerialization. Observations of the link between glycoproteins in the organic matrix and biomineralization in the parietal plates of the giant barnacle *A. psittacus* [45] and the proteomics data presented here suggest that the cuticle and adjacent tissues may play a role in deposition of adhesive material at the interface and biomineralization of the base plate. If so, the basal organic matrix is an important driver of the formation of barnacle cement and overall shell formation. In general, our proteomic analysis of these two matrix materials sheds light on how *A. amphitrite* has evolved to use the biochemical machinery of arthropods to thrive as a sessile organism in the harsh marine environment, thus offering clues as to how next-generation coatings could inhibit and counter barnacle biofouling on surfaces of interest.

## Data Availability

Mass spectrometry data (primary mass spectrometry data, intermediate peak lists, and formatted results) were deposited in the Mass Spectrometry Interactive Virtual Environment (MassIVE, http://massive.ucsd.edu), a member of the ProteomeXchange Consortium. The dataset was assigned MassIVE accession MSV000093654 [78]. Electronic supplementary material is available online [79].
